# How Do the First Days Count? A Case Study of Qatar Experience in Emergency Risk Communication during the MERS-CoV Outbreak

**DOI:** 10.3390/ijerph14121597

**Published:** 2017-12-19

**Authors:** Mohamed Nour, Mohd Alhajri, Elmoubasher A. B. A. Farag, Hamad E. Al-Romaihi, Mohamed Al-Thani, Salih Al-Marri, Elena Savoia

**Affiliations:** 1 Ministry of Public Health, Doha 11111, Qatar; malhajri1@moph.gov.qa (M.A.); eabdfarag@moph.gov.qa (E.A.B.A.F.); halromaihi@moph.gov.qa (H.E.A.-R.); malthani@moph.gov.qa (M.A.-T.); dralmarri@moph.gov.qa (S.A.-M.); 2 Division of Policy Translation & Leadership Development, Harvard T.H. Chan School of Public Health, Boston, MA 02115, USA

**Keywords:** MERS, emergency risk communication, coordination, Qatar, CERC, outbreak

## Abstract

This case study is the first to be developed in the Middle East region to document what happened during the response to the 2013 MERS outbreak in Qatar. It provides a description of key epidemiologic events and news released from a prime daily newspaper and main Emergency Risk Communication (ERC) actions that were undertaken by public health authorities. Using the Crisis and Emergency Risk Communication (CERC) theoretical framework, the study analyzes how the performed ERC strategies during the first days of the outbreak might have contributed to the outbreak management. Methods: MERS-CoV related events were chronologically tracked, together with the relevant stories that were published in a major newspaper over the course of three distinct phases of the epidemic. The collected media stories were then assessed against the practiced emergency risk communication (ERC) activities during the same time frame. Results: The Crisis & Emergency Risk Communication (CERC) framework was partially followed during the early days of the MERS-CoV epidemic, which were characterized by overwhelming uncertainty. The SCH’s commitment to a proactive and open risk communication strategy since day one, contributed to creating the SCH’s image as a credible source of information and allowed for the quick initiation of the overall response efforts. Yet, conflicting messages and over reassurance were among the observed pitfalls of the implemented ERC strategy. Conclusion: The adoption of CERC principles can help restore and maintain the credibility of responding agencies. Further work is needed to develop more rigorous and comprehensive research strategies that address sharing of information by mainstream as well as social media for a more accurate assessment of the impact of the ERC strategy.

## 1. Introduction

In May 2013, World Health Organization (WHO) Director-General Margaret Chan warned that a novel coronavirus, named Middle East Respiratory Syndrome Coronavirus (MERS-CoV), posed “a threat to the entire world” [1]. Since September 2012 to the time this case report was finalized (October 2017), WHO has received notification of 2090 laboratory-confirmed cases of MERS-CoV from 27 countries, including 730 deaths [2].

Since MERS-CoV is a relatively newly discovered virus, uncertainty regarding its epidemiological and clinical characteristics complicated the nature of the public health response to this epidemic [3]. Between 2012 and 2017, Qatar reported 19 MERS-CoV cases, including seven deaths.

Current scientific evidence suggests that dromedary camels are a major reservoir host for MERS-CoV and an animal source of transmission to humans [4]. Camel husbandry is a deeply rooted aspect of Qatari culture. Camels are deemed precious animals, a source of business and a symbol of socioeconomic status.

During emergency situations, the timely announcement, by public health authorities, of the first case of an infectious disease outbreak is important to establish and maintain public trust throughout the emergency [5,6,7]. Without securing such trust, authorities jeopardize epidemic control efforts and hence contribute to negative health, political, social, and economic consequences [8].

Nonetheless, authorities tend to be hesitant to declare an outbreak a public health emergency when there is insufficient information about its evolution and the effectiveness of recommended measures [5]. They are even more hesitant when an unfolding outbreak involves children, pets, or threatens livelihoods, and somehow impacts the social norms and political environment of a country or when there is a risk to expose organizational weaknesses in the handling of the situation [5]. The MERS-CoV epidemic was no exception to this rule.

This case study is the first to be developed in the Middle East region to provide a description of key epidemic developments along with key decisions in the implementation of the emergency risk communication (ERC) strategy and release of information by local print media.

We attempt to describe how the performed ERC strategy, during the first days of the outbreak, might have contributed to the authorities’ credibility, public trust, and outbreak control measures. We believe this case study might be useful to emergency planners for developing scenarios regarding what communication challenges to expect during the first days of a novel virus outbreak. It also represents an example of how practitioners, which are involved in the response to an outbreak, can engage in ERC evaluation efforts. Such efforts can be focused on ERC processes as in this case, or on ERC outcomes, and are both very much needed in public health emergency preparedness, as recently pointed by several literature reviews [9,10,11].

## 2. Theoretical Framework

The development of this case study is grounded on the Crisis and Emergency Risk Communication (CERC) framework, which was created by the U.S. Centers for Disease Control and Prevention (CDC) as a theoretical framework for research and practice [3,12]. Such framework describes how health communication functions within the context of risks and crisis. As the name reflects, CERC seeks to merge the traditions and best practices of risk communication into crisis communication. It positions communication centrally throughout the risk and crisis communication process, and it emphasizes the role of communication, primarily as a management tool from centralized authorities to the public through mass media channels. In this case report, we focus on the role of ERC as a crisis management tool during the MERS outbreak in Qatar by describing the delivery of information by central public health authorities and its reporting through a major media channel.

Evaluation is an integral part of the CERC framework. This case report is an attempt to apply the CERC framework in the conduction of evaluation activities that are aimed at monitoring key actions undertaken by the Supreme Council of Health (SCH) in Qatar during different phases of the epidemic and their relation to news reported by the media. The CERC lifecycle points to four phases: pre-crisis, initial, maintenance, and resolution. For each of these phases, specific types of information need to be created and delivered to the public. In the proposed case study we provide information on how the SCH followed some of the actions recommended in the CERC framework during the pre-crisis and initial phase of the 2013 MERS outbreak.

## 3. Methods

We have chronologically tracked Middle East Respiratory Syndrome Coronavirus (MERS-CoV) epidemiologic events and the Emergency Risk Communication (ERC) strategies implemented by the Supreme Council of Health (SCH) over the course of three phases of the epidemic (1st phase 23 September–19 November 2012; 2nd phase 20 November 2012–19 August 2013; 3rd phase 20 August 2013–17 March 2014) coupled with stories related to the MERS-CoV outbreak published in a major newspaper during the same time frame. These three phases were chosen because: (1) they represented the very critical time over the course of the epidemic where overwhelming uncertainty prevailed; (2) they were characterized by intensity of communication activities and media attention that accompanied the emergence of cases and/or the ensued public health response; (3) each phase roughly resembled the curve of an epidemic lifecycle (pre epidemic, epidemic, and post epidemic); and (4) each phase contained new breakthroughs or significant events that caused a remarkable turn in the course of the epidemic.

Our description is based on (1) data obtained from government documents and the reporting of other MERS-CoV outbreak major events by regional and international agencies and (2) stories related to MERS-CoV published in the local print media, namely in Al-Raya Newspaper.

Al-Raya was chosen because it was leading among the prime media outlets used by the SCH to publish updates on the epidemic and it was the source of media that massively covered the development of the epidemic (a total of 134 news stories were identified as follows: 108 news articles, 19 press releases, three press conferences reports, and two interviews). In Qatar, narratives across the local newspapers are largely the same, particularly in publishing official press releases. Al-Raya newspaper was leading daily in terms of readership compared to the other four Arabic dailies in Qatar based on circulation statistics. As such, we believe that in Qatar, Al-Raya newspaper can be fairly reflective of the mainstream media. However, we acknowledge that there is a limitation in assessing only one source of print media and remark that this study does not intend to report a comprehensive media content analysis that exhausts all types of media.

An electronic search was run for the word “corona” in the online archive of Al-Raya Newspaper to retrieve all of the relevant news stories. The extraction of news articles was performed by four research team members who independently reviewed each article and performed a content analysis of the selected news stories, comparing results, and discussing disagreements. These stories were then compiled and matched with the SCH’s press releases, records of activities performed by the national response authorities, and public reactions, in addition to the “rumors” detected by the Governmental Health Communication Centre within the Supreme Council of Health (SCH), in Qatar through monitoring of MERS related news.

## 4. Results

### 4.1. Pre-Epidemic Phase

During the emergency preparedness planning phase, the Supreme Council of Health (SCH) and the Animal Health Department (AHD) agreed to establish a joint leadership response to the Middle East Respiratory Syndrome Coronavirus (MERS-CoV) outbreak, including a unified communication approach. In Qatar, public health actions that were taken in emergency situations are based on the National Early Preparedness and Response (EPR) plan, which is guided by the International Health Regulations (IHR 2005). Qatar’s EPR plan states that “*any verified public health event of national and/or international concern will transparently be announced*”. In keeping with this regulation, designated spokespersons were identified in the plan to be in charge of delivering messages to the public. The plan states that all of the messages need to be jointly approved by the SCH and AHD. Such actions are aligned with the pre-crisis actions that are underlined in the Crisis Emergency Risk Communication (CERC) framework, more specifically with the importance of preparing, fostering alliances, and develop consensus.

### 4.2. First Phase of the Epidemic (23 September to 19 November), 2012

The emergence of the first case as Hajj season was approaching: descriptions of key epidemic events, actions taken by the SCH and other key stakeholders, as well as key news reports are provided and summarized in Table 1.

23 September 2012: On this date, the first laboratory case of MERS-CoV in Qatar (Q1) was confirmed by United Kingdom (UK) health authorities [5]. A press conference was held the following day, 24 September 2012, by the SCH’s designated spokesperson, during which the case was confirmed. This action was aligned with the CERC framework to provide the information to the public as soon as possible, and have a designated spokesperson do so. During the press conference, the SCH admitted the lack of knowledge on how to best clinically manage the case. This action was also consistent with the CERC framework recommendation of acknowledging the uncertainty of the situation. While acknowledging the lack of clinical experience, emphasis was placed on the extensive efforts that the medical team undertook in attempting to treat the patient. During the press conference, the SCH also emphasized that there was “*no need to panic*”, and explained that the surveillance system had been reinforced nationwide and was capable of identifying other cases. This action is aligned with the CERC framework recommendation of expressing that a process is in place. Simultaneously, Al-Raya newspaper published a full report detailing what was known about the disease, as sourced from the WHO website. 

Public health officials at the SCH, including members of the EPR team, engaged in intensive consultations with WHO and the Health Protection Agency (HPA) in the United Kingdom (UK) over the three days that followed the first confirmed case. The primary purpose of these consultations was to share information including: providing updates on the clinical status of the patient, assessing the potential risk for the spread of the disease, and informing the decisions regarding the national alertness level and appropriate disease control strategies, as based on a set of potential scenarios of how the outbreak could evolve. This action was in accordance with the CERC framework recommendation of coordinating and collaborating with credible sources.

Meanwhile, media interest was growing. The condition of the afflicted patient was making headlines on the front pages of local newspapers. Journalists established a direct communication channel with a relative of the patient and clinical updates were regularly published over the course of the week. The patient’s relative expressed dissatisfaction with the medical care provided, and ultimately opted to transfer the patient to the UK. The family acknowledged that they had received support from the Emir, who covered the medical expenses abroad, and that the SCH was in contact with the UK hospital to which the patient was admitted to get frequent updates on the clinical status of the patient. At this point in time, Al-Raya newspaper reported that no new cases were identified and that preventive measures had been initiated.

24 September–2 October 2012. The timing of this outbreak, approximately three weeks prior to the Hajj season (24–29 October), generated interest in the media because of the potential risk of the virus spreading among pilgrims. Media reports focused on updates on the epidemic, on the preventive measures and on a WHO report that acknowledged the severity of the disease but communicated that there was no intention to impose restrictions on travel and trade, in alignment with the Kingdom of Saudi Arabia’s (KSA) official policy.

More specifically, media reports described the outbreak by focusing on three different topics: (1) the role of the SCH in receiving updates on the clinical situation of the patient transferred to the UK; (2) the potential risk of greater spread of the outbreak during the approaching Hajj season; and (3) similarities between the virus causing MERS and the one causing the Severe Acute Respiratory Syndrome (SARS).

A press release was issued by the SCH three days after the detection of a rumor saying that the SCH did not undertake appropriate efforts to support the transfer of the patient to the UK. The press release was timed for weekday release, in order to reach a larger readership, and it detailed how the SCH was monitoring the patient’s condition. This action is aligned with the CERC framework recommendation of monitoring rumors and dispelling them quickly. In order to address the fears regarding the risk of spread during the Hajj season starting on 24 October, the KSA Minister of Health released a statement emphasizing that, in the regional and religious context of the evolving outbreak, the situation was not of particular concern, as the novel virus did not seem to spread easily among humans. A SCH spokesperson was interviewed regarding the preparations for the Hajj on 3 October 2012. During the interview, the spokesperson reassured the public by focusing on the following two facts: (1) seasonal flu immunization is mandatory, as per KSA health regulations for pilgrims; and that (2) the situation was being monitored through information sharing with the KSA and WHO.

### 4.3. Actions of the ERC Network of Decision Makers, Professional Partners, and Interested Parties

A previous study that was conducted in South Korea during the 2015 MERS outbreak suggests that interagency risk communication strategies are important means to improve interagency effort toward a virus outbreak [13]. We describe below the network of agencies that are engaged in the ERC strategy in Qatar.

The National Outbreak Control Taskforce (NOCT): There was an urgent need for information to support the decision-making process of the NOCT, which was formed by the SCH to respond to the outbreak. As there were only a small number of reported MERS-CoV cases, the main information sources used by the decision-makers were WHO, KSA, and HPA in the UK, and the media. This information served as a basis for the development of the risk assessment plan and the roadmap for a response. The roadmap was developed by WHO and the Food and Agriculture Organization of the United Nations (FAO) providing technical support in response to Qatar’s request. The roadmap emphasized: enhanced surveillance; case management, isolation, infection prevention and control; health education; and, risk communication. Despite these efforts, public fear was palpable due to the approaching Hajj season. Therefore, a significant part of the technical discussion that involved the Medical Committee for the Hajj focused on considering potential epidemic scenarios.

Assessing the potential risk was a critical component of the WHO support, as the roadmap that it helped develop eased securing the resources required for investigation and response measures. Thus, decision makers approved the proposed roadmap despite the overwhelming uncertainty regarding both the nature of the epidemic and the indefinite outcomes of the ratified proposed actions.

Technical meetings and exchange of emails constituted the primary mechanisms for information sharing between the NOCT network and relevant stakeholders. Since the beginning of the epidemic, daily and weekly surveillance reports were communicated among the NOCT and the leaders of the represented institutions as part of stakeholders’ information sharing, consistent with the CERC recommendations. Thanks to such reports, public health experts were able to respond quickly to requests for clarification on surveillance. This allowed for a national consensus on the anticipated risk of MERS-CoV and the establishment of a recommended course of action, and it ensured a collective understanding among risk communicators, minimizing the amount of contradictory messages given to the public.

The ERC strategy included community engagement in order to facilitate the sharing of information to the public and persuades community-based organizations, including the Camel Race Association, to participate in investigation efforts. This action is aligned with the CERC framework recommendation of engaging at-risk population in the ERC strategy. For example, epidemiologic investigations necessitated the engagement of people at risk (some of whom had no symptoms) in practicing the isolation of suspected cases at the hospital for three to five days until receipt of laboratory results, and in the screening of contacts. Such screening typically implies listing all relatives, friends, and coworkers who were believed to have come in contact with the suspected case to be tested. Until the laboratory results were unveiled, such contacts experienced tension and worries of being infected.

Registration process to the Hajj: Before registering for the Hajj, prospective pilgrims were advised to check their health and vaccination status. The elderly, those with co-morbidities, and people with a compromised immune system were denied registration, as these groups were believed to be the most vulnerable to the novel virus. Religious Affairs echoed the public health message and reiterated the recommendation to take appropriate precautions when participating in the Hajj. All of the medical and public health preparations, including coordination with the KSA health authorities, were regularly communicated with the public. Compliance of the prospective pilgrims with the recommended actions was close to 100%.

Role of healthcare workers: As part of the response, healthcare workers (HCWs) received training on what was known about the novel virus. The training informed HCWs on the evolving epidemiological situation, the updated WHO risk assessment, the appropriate infection prevention and control measures, and their role within the national response system. Staff working at Emergency Room Departments (ERD), isolation rooms, and intensive care units were prioritized to receive the training and were asked to share their knowledge with colleagues. Regular reminders about Infection Prevention and Control (IPC) were sent to the HCWs at Infectious Diseases departments across healthcare facilities in Qatar. Each HCW was viewed as playing a critical role in information sharing within their public networks, and, thusly, each HCW received regular updates on the situation and the national response via e-mail. IPC measures were strictly monitored, and thanks to the triage policy, suspected cases were rapidly identified, isolated, and reported. 

HCWs were engaged in communicating the risk with patients and their families. Whenever a suspected case was identified as a Qatari national, a Qatari senior physician was called upon to explain the protocol of medical isolation, the medical and public health investigation procedures, and the expected prognosis. This policy was adopted to ensure greater openness, trust, and collaboration. Consequently, neither refusal nor lack of compliance with established control measures, including isolation, were reported, even as a greater number of cases were identified.

On 4 October 2012, the media reported that the first Qatari case (Q1) had been cured and that the patient was recovering. The media also reported on a press conference held by the Medical Committee of the Qatar Hajj Commission stating that “*all clinical and preventive preparations for the Hajj season were in place and that there was no concern of the emergence of an outbreak as no scientific evidence was available on human-to-human transmission up to that point in time*”. Despite the concern, no cases of the novel coronavirus were reported among the 3.2 million pilgrims, the citizens of the KSA, or the citizens of Qatar until after the end of October 2012.

### 4.4. Second Phase of the Epidemic (20 November 2012 to 19 August 2013): Reporting of Second Qatari Case as Further Cases Including Deaths Continued to Be Reproted in the Region: A Description of Key Epidemic Events, Actions Taken by the SCH and other Key Stakeholders as Well as Key News Reports Is Provided Below and Summarized in Table 2

On 24 November 2012, the SCH issued a press release reporting that a second case of the novel virus had been confirmed. The press release was issued after four days of the case confirmation. It stated the following: (1) the patient was admitted to the hospital by the end of October and was diagnosed with the novel virus on 20 November; (2) the patient was recovering but had been transferred abroad upon the request of his family; (3) all of the patient’s suspected contacts were screened and tested negative as confirmed by a qualified external laboratory; (4) WHO had been officially notified of the case (Q2), which was identified as the sixth case worldwide; (5) intensive consultations were held via conference call with several scientific entities (such as WHO) on the day following the confirmation of the case (21 November). The media reacted by circulating a WHO report on the disease remarking that MERS-CoV belongs to the SARS family and that an alert was issued to reinforce surveillance globally. However, it acknowledged that more information was needed to understand MERS-CoV’s virology (Table 2).

At this point in time, the epidemiological investigation suggested a hypothesis on the possible animal source of the contagion. It was reported that the last patient was taken to the hospital directly from his camel barn. Subsequently, it was acknowledged that two of the six known cases had some exposure to camels. A decision was made by the NOCT to visit the patient’s camel barn and interview his potential contacts. This visit was challenging for both the field investigation team and the camel owners. While the owners were concerned about the possible social stigma, the visiting investigation team members were concerned about the possible risk of becoming infected.

On 25 November 2012, the media portrayed the public in fear and demanding a scaling up of the healthcare response in terms of the following: (1) raising public awareness about the virus; (2) enhancing the surveillance system to monitor the situation more vigilantly to promptly detect any possible case; and (3) developing advanced diagnostic and treatment capacities to enable the prompt confirmation of suspected cases and offer an optimal medical care domestically, with the implied enhancement of training of medical staff. The media quoted a senator stating that “*I will file an urgent request to discuss the disease in the upcoming senate session*”. A healthcare professional from Hamad General Hospital (HGH), the national referral hospital, also reported: “*as of now no evidence indicating that the situation is worrisome and the disease does not seem to be outbreak-prone according to the WHO, so no need to panic. I think authorities will not hesitate to take all required measures if the risk proved to be genuine*”. The Newspapers persistently framed the novel virus as being as fatal as SARS, as both belonging to the same family of coronaviruses and presenting with similar clinical features.

On 27 November 2012, the SCH disseminated a press release disclosing the minutes from a meeting that detailed the response efforts that were undertaken upon the confirmation of the second case. The press release proclaimed that most of the measures that the public demanded were already in place and that consultation with WHO was ongoing. The media accounts provided basic information about the disease but mostly focused on the personal story of the second Qatari patient.

### 4.5. Third Phase of the Epidemic (20 August 2013–17 March 2014): Fresh Qatari Cases and Deaths Reported and the Isolation of the Virus from Camels: A Description of Key Epidemic Events, Actions Taken by the SCH and Other Key Stakeholders as Well as Key News Reports Is Provided Below and Summarized in Table 3

Between November 2012 (end of the Second Phase reported above) and August 2013, the KSA continued to report cases and deaths caused by MERS-CoV. The hypothesis that MERS-CoV infection was related to camels resurfaced when the local media covered a British study suggesting that camels may have a possible role in MERS-CoV transmission to humans. This finding was considered very controversial because of the high consideration with which camels are held in Qatar society (Table 3).

On 20 August 2013, the SCH published a press release stating that a third MERS-CoV case (Q3) had been confirmed. This third patient was a Qatari national who was diagnosed abroad and his clinical was reported as not severe and stable. The press release re-emphasized that the authorities were aware of, and responding to, public demands, and stated that monitoring of all the acute respiratory illnesses was ongoing, the diagnostic capacity was locally available, and that the SCH had established a helpline for the public and created a dedicated webpage to allow for up to date information about the situation. 

On 22 August 2013, the SCH launched an awareness campaign on MERS-CoV, which publicized the newly established webpage and helpline services. This campaign coincided with the Hajj season during the second year of the epidemic. 

By 27 August 2013, the SCH announced in a press release that another new MERS-CoV case had been detected in Qatar (Q4) and that the patient was in critical condition. The newly detected case had been in contact with a previously confirmed MERS-CoV case and had comorbidities that were aggravating factors for his condition. 

On 7 September 2013, the newly confirmed MERS-CoV case (Q4), who had previously been in critical condition, was declared dead and the antecedent case (Q3) was announced as cured. Meanwhile, MERS-CoV cases were still being reported in the KSA, and the public feared that the disease could spread during the approaching Hajj season. In order to respond to the concerns of the public, a senior SCH spokesperson held an interview on 29 September 2013. During the interview, the spokesperson reiterated the preventive measures and the preparation efforts that were undertaken by the government, and provided recommendations for ensuring the safety of Qatari pilgrims. The spokesperson also provided the public with the most up-to-date information on MERS-CoV.

On 18 October 2013, the SCH proclaimed that they had detected a new MERS-CoV case (Q5) another Qatari citizen. A statement from the KSA health authorities was published in a local newspaper reporting that “*Hajj season had passed without reporting a single MERS-CoV case among pilgrims*”. However, MERS-CoV cases continued to be reported from other parts of the KSA after the Hajj was concluded. The total number of reported cases reached 120, among which 51 perished.

On 27 October 2013, the SCH revealed, in a press release, that a new MERS-CoV case had been detected (Q6). The patient was symptom-free but had been in contact with a subject, who was a laboratory-confirmed case. The press release also reported that the previously declared case (Q5) had been cured and discharged from the hospital. The Sultanate of Oman declared its first confirmed MERS-CoV case and the KSA continuously reported new cases, with casualties mounting to 53 deaths; no new case was reported in Qatar until 8 November 2013. At this point in time, school principals in Qatar voiced their concern that there was an insufficient number of school nurses to meet the course of action recommended by the health authorities. It was only seven days until another MERS-CoV case (Q7) was confirmed in Qatar in a patient with chronic illnesses.

By 13 November 2013, the State of Kuwait also reported two cases, one of which had just returned from the KSA, while the Sultanate of Oman reported its first MERS-CoV related death. The KSA then released a report confirming that the virus had been isolated in seven camels, bringing the zoonotic nature of the disease to light for the first time and Qatar reported the death of two MERS-CoV cases on 19 and 21 November 2013, respectively, bringing the total MERS-CoV related deaths in the country to three, whereas the number of those killed by the virus in KSA reached 55 out of 130 reported cases. 

On 25 November 2013, the Qatari national senate held a special debriefing session to discuss the MERS-CoV epidemic and the prevention and control measures being undertaken by the Qatari government. Media coverage of the event highlighted the senators’ demand for a reinforced national prevention and control strategy. 

On 28 November 2013, the SCH announced that the virus was isolated from two camels in a barn where two of the previously confirmed human cases were reported. This news coverage attracted the attention of the camel owners who when interviewed by the media, criticized the Ministry of Environment (MOE) for the lack of proactive measures, and for withholding information.

On 2 December 2013, SCH and MOE organized a joint press conference to address the concerns of the public. The organizations hoped to disseminate the messages, that, firstly, the MERS-CoV epidemic in Qatar was not spreading, and, secondly, the recent detection of the source of the virus would have contributed to a greater understanding of the nature of the disease and its mode of transmission. A national plan to screen camels was announced; nonetheless, the SCH and MOE explained that there was no intention to restrict camel movement, due to the fact that there was insufficient evidence of the role camels played in the disease transmission pattern. 

On 6 December 2013, the MOE stated that Qatar is “safe” from the virus, as ongoing screening was being jointly performed with SCH.

On 17 March 2014, Qatar announced the epidemic was controlled in camels. No other significant events occurred and no further new cases were reported in the country until November 2014. Nevertheless, confronting MERS-CoV remained a top public priority, as cases and deaths continued to be reported in neighboring countries.

## 5. Discussion

This case report aims to document what happened during the response to the MERS outbreak in Qatar by describing the sequence of epidemiologic events and news that was derived by the monitoring of articles from “Al-Raya newspaper” in regards to the emergence and development of the outbreak and main actions undertaken by the SCH to communicate to the public. This case study presents a basic evaluation effort of the ERC strategy adopted by the SCH during the 2013 MERS-CoV outbreak analyzed by the use of the CERC framework. Actions that were undertaken by the SCH have been described and related to recommendations that were outlined in the CERC framework when appropriate. 

Despite the overwhelming uncertainty during the early days of the MERS-CoV epidemic, the SCH’s commitment to a proactive and open risk communication strategy, which included the sharing of information in a timely manner “through prearranged channels” on new cases and deaths, and acknowledging the lack of experience in responding to the situation, supported the SCH’s image as a credible source of information and allowed for the quick initiation of overall response efforts. Such actions are well aligned with recommendations that were provided by the CERC framework. We believe that the acknowledgment of uncertainty since the beginning of the response allowed the SCH to increase public attention and situational awareness among responding agencies; and, that close consultation with international institutions of reference (i.e., WHO) contributed to the image of a competent governance and a unified communication strategy. By doing so, the authorities not only acknowledged the situation but also reported actions undertaken (e.g., investigations and epidemiological studies) and worked to achieve credibility by initiating a quick response to rumors and misperceptions.

However, the response was not without error. The inherent uncertainty and lack of sufficient information caused the government spokespersons and the media to provide conflicting messages, which might have contributed to raising public concern. For example, on the very first press conference held on 24 September 2012, the spokesperson called on the public “*not to panic*”, despite the unknown path the “*novel virus*” could have taken. As a consequence, the media persistently focused on the resemblance of MERS-CoV to SARS to anchor the lack of information on previous knowledge and experience [3]. Similarly, another public official announced, on 6 December 2013, (after almost one year from the first case) that Qatar is “*safe*”, which seemed to contradict the fact that MERS-CoV’s mode of transmission remained unclear and neighboring countries continued to report new cases and deaths.

Overall, based on our experience in responding to this outbreak, we believe that having trained and experienced individuals assigned as spokespersons, as recommended by the CERC framework, positively contributed to the ERCs activities, as these spokespersons were prepared to respond to questions from the media, and, in doing so, they helped to maintain the image of the SCH as an accountable and credible organization, and to the extent possible, minimized the chance for conflicting messages. 

Agreements between the SCH and other organizations to have one-national unified message further bolstered a unified official response. The involved parties, particularly MOE and Ministry of Municipalities (MOM), were able to share information as it evolved (as recommended by the CERC framework in relation to stakeholders communication) and make decisions that were based on the previously agreed upon roles and responsibilities as stated in the national emergency plan. Similarly, in the healthcare setting, physicians were kept informed on the situation, and, as a consequence, are able to openly explain the nature of the disease and discuss its clinical prognosis with MERS-CoV patients and their families. 

Our analysis has several limitations: the data rely on one source of mainstream print media. Future research should focus on analyzing information shared on social media by the Qatari authorities and the public. As communication activities were not based on audience analysis and media preferences, we acknowledge that it is difficult to assess the effectiveness of the message in a conclusive way especially in regards to adopted behaviors. Yet, public compliance with public health measures could be demonstrated through several anecdotal ways, including the acceptance of individuals considered suspected cases to be isolated and tested, a willingness of those with symptoms to be hospitalized, as well as the openness of camel owners to join the MERS-CoV field investigation teams visiting their barns. 

## 6. Conclusions

This manuscript describes the key events that occurred during the response to the MERS outbreak in Qatar by using government documents and media monitoring. This constitutes a basic monitoring effort to document the SCH’s ERC strategy during the 2013 MERS-CoV outbreak.

Based on the proposed analysis, we believe that SCH’s early preparedness to the epidemic and its commitment to a proactive and open risk communication strategy, which included actions during the first days of the outbreak such as sharing of information in a timely manner with the public and domestic and international organizations, and acknowledging the uncertainty of the situation, favored the establishment of the SCH’s image as a credible source of information and allowed for the quick initiation of overall response efforts and effective rumor control during the course of the outbreak. Moreover, despite that the CERC framework was only partially adopted in Qatar, this case study consolidated the validity of the framework and also emphasized the significant role of communication primarily as a public health tool for managing emergencies.

Yet, further work is needed to develop more rigorous and comprehensive research strategies to assess the impact of ERC during MERS or any other emergency situation in light of such framework. Use of social media and other channels of communication should be the focus of future research activities, as well as the impact of information sharing and institutional collective action on ERC effectiveness during similar crises.

## Figures and Tables

**Table 1:** First phase of the epidemic (23 September–19 November).

Date	Epidemic Events and Other Key Public Health Actions	Executed SCH Communication Activities and Key Messages	Al-Raya Newspaper Reports and Key Messages	Assessment Remarks Based on Crisis Emergency Risk Communication (CERC)
23 September 2012	The first laboratory confirmed case is declared in Britain			A novel and virus with great deal of uncertainty. A typical situation at the outset of an emerging virus.
24 September 2012	Surveillance system was reinforced. Technical Consultations with WHO & HPA in the UK were initiated	Press conference: Supreme Council of Health (SCH) confirmed the first case admitting lack of knowledge on how to best clinically manage the case. No need to “Panic”, as no further cases were detected.		Despite the scarce information and the overwhelming uncertainty, the SCH managed to timely announce the case and explain the undertaken measures, in line with the CERC which calls for admitting the limitations while telling what actions are being done to bring answers (consultations with the WHO). This constitutes the ‘initial phase’ as described by the CERC. However, empathy was hardly expressed to the patient and his family - it was a little early to generate behavioral messages that conveyed a paternalistic approach (without reality check). These were largely not in accordance with the CERC.
25 September 2012	National Outbreak Control Taskforce (NOCT) was activated		Reports on the information shared during the press conference, adding facts about Middle East Respiratory Syndrome Coronavirus (MERS) as sourced from the WHO website. The SCH was blamed for not providing sufficient follow up to the patient.	The Qatari early preparedness based on the previous experience with the SARS and H1N1 helped activate the national response in relatively short time (stakeholder communication and coordination). While CERC stated that people and media would eagerly seek for other sources of information during emergency situations, the SCH seemed not to have considered this behavior as it did not provide a sustained flow of information, allowing for the local media to blame the sluggish response.
26–27 September 2012	SCH Maintains close communication with the UK hospital where the patient was transferred to be abreast of the clinical developments of the patient		Reports on patient status and transfer to the UK: Family members were interviewed. SCH response was described as “late” and that the authorities only come to know about the case from the international media and the WHO. Reports on the potential risk of greater spread during the approaching Hajj, and Reports on similarities between MERS and Severe Acute Respiratory Syndrome (SARS).	Despite initiating reasonable public health measures, the SCH still seemed not to cope with the urgency of the situation. SCH should have publicized the ongoing efforts at the different levels including the persistent follow up of the patient.
29 September 2012	Development of the risk assessment plan and roadmap to response with (with support from WHO and FAO)	Press Release: The patient is improving and the SCH was engaged with the UK authorities to closely monitor the patient. This is in response to the rumor that the Qatari authorities are not providing the care to the case.	Reports on patient status and transfer to the UK: Family members are interviewed. They suggested that a medical specialist should have been sent to UK to accompany the patient.	Although it has come a little late, the SCH reacted to the rumor with a press release but it should have further displayed the progression on the technical efforts with the WHO and FAO to guide the public health response.
1 October 2012	Health preparations for Hajj season were initiated: Seasonal flu vaccine is available			The approaching Hajj season heightened the fears of the public that MERS might spread widely during the big gathering event. This constituted another test to the readiness and the nature of coordination of the authorities at the local and the regional levels. The displayed aspects included: provision of the Flu vaccine (the preventive role of which is yet to be confirmed), besides the pre-travel medical screening and education.
2 October 2012	Coordination with stakeholders was maintained	Press Release: As a new virus, it was hard even for an advanced country like the UK to discover the nature of the novel virus until late.		Again the messages were not developed according to CERC recommendations. It could have better explain the progression on the preparedness efforts and help shift the public focus to things to do.
3 October 2012	Healthcare Workers training implemented	SCH spoke-person is interviewed. Hajj preparations considered Flu and the risk of MERS.	The media reported on the interview which emphasized that: Seasonal flu immunization is mandatory, as per KSA health regulations for pilgrims, and that the health situation was being monitored through information sharing with the KSA and WHO.	The spokesperson seemed to have answered some of the key current questions. While the media focused on Hajj preparations, SCH should have bring the attention to the reinforcement of the preparations (training of the healthcare workers).
4 October 2012		Press conference of the Medical Committee of the Qatar Hajj Commission. Various epidemic scenarios were considered.	The media reported on the press conference which addressed the clinical and preventive measures including the risk of MERS. Reports on first MERS case being cured.	The fact that other authorities counted for the new virus in their preparation package to the Hajj season indicated the level of coordination among stakeholders.
5 October–19 November 2012	Coordination between the KSA and Qatar authorities during Hajj. Close monitoring of the situation with support from WHO	Focus on Hajj preventive measures.	Reports focused on Hajj seasons: public concern of the potential spread of the disease during the season.	Here the authorities indicated their international level of coordination for Hajj with the relevant authorities.
Pilgrimage Season—in 2012 the end of the Hajj season was on 24–29 October. However, pilgrims start to travel to Saudi Arabia 8–10 weeks prior to that. Therefore since the inception of the first case, Qatar was experiencing pilgrimage season.				

CERC: Crisis Emergency Risk Communication; SCH: Supreme Council of Health; MERS: Middle East Respiratory Syndrome Coronavirus.

**Table 2:** Second phase of the epidemic (20 November 2012–August 2013).

Date	Epidemic Events and Other Key Public Health Actions	Executed SCH Communication Activities and Key Messages	Al-Raya Newspaper Reports and Key Messages	Assessment Remarks Based on CERC
20 November 2012	Second case confirmed in Qatar (6th case worldwide)			The possibility to have fresh cases was always there, providing a chance to test the recently activated public health preparedness and response including the communication strategies as recommended by CERC.
21 November 2012	WHO notified			
24 November 2012	Intensive technical consultations with the WHO	Press release focused on confirmation of the second MERS case besides reporting on the SCH actions: - Patient transferred to the UK - Contact tracing - Collaboration with WHO in place	Reports focused on second case and similarities between MERS and SARS as reported by WHO	The second case was not immediately declared despite it was reported to the WHO on time. Yet, the SCH press release served the purposes of informing the public with the key details besides the executed measures in response to it. Highlighting the communication with the WHO seemed to help restore the public trust: reliance on the consultations with the WHO to make the appropriate decisions.
25 November 2012	Exposure to camels as a potential source of the infection being hypothesized NOTC decides to visit camel barns as part of the field investigation despite concerns of risk of infection		The media reported the second Qatari case and reflected the public fears and demands to bring about an enhanced response in addition to the capacity to diagnose cases domestically Senators and healthcare providers being interviewed MERS portrayed as fatal as SARS Camel owners concerned with potential social stigma	Persistent media attention. The media invested in the novelty of the threat to raise the public concern and thereby, poking the SCH (via telling public demands) to step up its response efforts. This might either indicate that the public was not made fully aware of the ongoing response efforts or the overall response was not yet up to the expected level (the local capacity to laboratory confirm cases then treat them was not there yet). Nonetheless, the media attention seemed to help initiate a perception of the virus potential risk.
27 November 2012	Consultation with WHO ongoing MERS Cases and deaths continued to be reported from KSA	SCH press release confirming that most of the measures requested by the public were already in place	Reports on personal story of the second Qatari case	While this created an opportunity to display competencies, it allowed the media (and the public) to score a triumph of being one step ahead of the SCH by raising public demands to bring about more resources and enhance the overall response. The fact that cases and deaths are also being reported from the other neighboring countries in the region might seem to help accept the risk which is yet an exotic one but now looks natural and fairly distributed.

Cases and deaths continued to be reported from KSA (Kingdom of Saudi Arabia) through August 2013.

**Table 3:** Third phase of the epidemic (20 August 2013–17 March 2014).

Date	Epidemic Events and Other Key Public Health Actions	Executed SCH Communication Activities and Key Messages	Al-Raya Newspaper Reports and Key Messages	Assessment Remarks Based on CERC
20 August 2013	Third case confirmed Hajj season pilgrimage begins Communication with major stakeholders is maintained including neighboring countries	Press release focused on: (1) Third case confirmed (2) Surveillance in place (3) Webpage and helpline being available Public awareness campaign launched	Reports on concerns related to forthcoming Hajj season. Local media reports a British study tells that camels might have a role in MERS transmission Media reported on the press release, stating that diagnostic capacity was made available.	This phase largely corresponds to the ‘maintenance phase of CERC’. After a 9 months pause, a new MERS case was reported in Qatar. Despite no cases, in contrary to the initial expectations pertaining the virus behavior, the media fairly occupied the vacuum by reporting on the disease cases and deaths reported by the other neighboring countries in the region. Having no cases might indicate either there were no cases for real or the surveillance system was poor (unable to detect cases). The SCH refuted the latter emphasizing the alertness of the surveillance system. The public need for access to further information seemed to be appreciated as a specific webpage was introduced. This action came as a response to the public demands. Yet, it is unclear whether the awareness campaign has just coincided with the new case or it was launched as an upbringing of the national response.
22 August 2013	SCH launches an awareness campaign including helpline services		The media reported on the awareness campaign and helpline service.	The campaign seemed to have helped the public get better understanding of the virus threat as more information now was made accessible. The campaign also created a good chance to provide facts, refute rumors and correct misperceptions. But it is unclear whether the campaign was designed based on CERC principles and whether it was followed by assessment.
27 August 2013	Fourth case confirmed as contact of previous case	Press release announcing the fourth case (patient with comorbidities)		The prime event is the death of a MERS confirmed case. The SCH decided to declare this death coupled with the cure of another recently confirmed case.
7 September 2013	Fourth case dies and third case is cured	Press release announcing the death of the fourth case and recovery of the third case		This press release seemed to have conveyed two messages; while it acknowledged the death of an infected person, it declared the other was free of the infection and discharged home, indirectly reassuring the public that not all of those who contract the infection will eventually end up dead. The local healthcare system could be still be trusted.
29 September 2013		Interview with SCH spoke-person focused on: Preventive measures for Hajj season Recommendations for Qatari pilgrims Updated information on MERS		The second Hajj season since the start of the epidemic. Despite the previous season passed uneventful in terms of virus spread, concerns remained high that it can still affect Pilgrims. This seemed to have necessitated the SCH sharing of the preparedness measures and coordination between Health and other sectors besides coordination between Qatar and KSA authorities.
18 October 2013	Fifth case confirmed End of Hajj season	Press release announcing the fifth case	Reports on absence of causes related to the Hajj season based on a statement from KSA authorities.	While declaring Hajj season is over, the fifth case was proclaimed, in line with the SCH policy of transparency. As part of the heightened media and public attention, MERS news continued to be newsworthy. Nevertheless, epidemiologists were unable to explain why the virus did not spread in the Hajj big gathering.
27 October 2013	Sixth case confirmed The Sultanate of Omar announces the first case KSA continues to report cases	Press release announcing the sixth case (contact of previous case), fifth case cured.	Some of the recently reported cases and deaths were from the eastern part of KSA (sharing borders with Qatar). KSA was the biggest affected country so far.	More facts were being shared to help establish a realistic understanding of the virus. The press release explained that human-to-human transmission was possible but it also consolidated that it could be treated (locally).
3 November 2013	Seventh case confirmed	Press release announcing the seventh case.		There were concerns now as to why after 9 months of zero cases people begin to hear about new 5 cases being reported in less than 2 months. This created more pressure on the SCH professionals to bring about reasonable explanations.
13 November 2013	The Sultanate of Omar announces that the first case died Kuwait reports two cases KSA reports the virus was detected in camels		Media kept reporting the spread of MERS in the Gulf Countries.	Reporting of new MERS cases from countries across the region seemed to have helped the public accept the virus risk as a fairly distributed one. But the virus detection in camels was a key turning point of the epidemic course. Camels are involved in peoples' lives (socioeconomically) across the Arab Peninsula, making it seriously challenging to investigation efforts.
19–21 November 2013	Death of 2 cases (two days apart) KSA—55 deaths and 130 reported cases	Press release announcing the death of two cases on 19 and 21 November respectively (total 3 deaths in the country).	Those who died were either suffering a chronic illness already or had an immunosuppressing condition.	Reporting more deaths related to MERS and acknowledging the fatality nature of the virus. However, a simple comparison with KSA was reassuring to the local community in Qatar.
25 November 2013	Special debriefing from the Qatari national senate focused on prevention and control measures	The senate recommendation acknowledged the ongoing investigation and control efforts but demanded these to be reinforced in terms of: disease monitoring domestically and abroad; expansion of isolation capacity in the healthcare facilities; screening of humans and animals.	Reports focused on senators’ demand for reinforced preventive measures. The senate demands were somehow portrayed as a response to the media outcry to step up the national response.	Since the break out of the first MERS case, this is considered the second remarkable call on SCH and its partners to boost the national preparedness and response to the virus threat. Despite the situation in the country was not as bad as it was in other neighboring countries, but having the subject discussed in the senate could indicate to serious public concerns about the quality of the SCH response. Further, it might indicate the need to make the ongoing investigation and control efforts (besides the available competencies) more visible. Nonetheless, the senate session seemed to have helped secure more resources and support to the ongoing control efforts.
28 November 2013	MERS virus was isolated from camels in Qatar	Press release: SCH announced the virus was isolated from two camels.	Reports on interviews with camel owners criticizing the Ministry of Environment (MOE) for the lack of proactive measures and for withholding information.	This epidemiological breakthrough helped proof that SCH was not waiting passively for the big technical institutions to reveal the virus characteristic but rather was really engaged with them in the efforts to understand the disease characteristic and risk. Yet, this discovery also implied that the investigation efforts would not be easy. It would not be easy to convince the camel owners (with the majority involved in the camel race business) to accept the notion that their camels might play a role in the disease transmission.
2–6 December 2013		SCH and MOE joint press conference to address the concern of the public and camel owners focused on: (1) The status of the outbreak (2) Efforts to contain the epidemic (3) The recent discovery (the virus isolation from the camels) as a possible progression towards the control of the epidemic (4) Screening of camels will be initiated soon (5) No plans to impose restrictions on trade as of now	The print media neutrally reported the messages which were communicated during the press conference.	SCH and its main partner (Animal Health Department) decided to hold a press conference as a form of communicating the new updates related to MERS, instead of issuing press release. For the second time, one of the spokespersons reassured that "the situation is stable" while yet more is to be known about the virus risk and behavior. This was not in accordance with the CERC recommendations. It seems that the press conference was a mean to prepare the public to collaborate with the intended national screening surveys. The grounds on which the selection of the communication method was unclear.
6 December 2013–17 March 2014	Animal Health Department announced control of the epidemic in camels as no new human case reported.		Qatar Animal resources are secured from the “leak” of coronavirus. Camels will be subjected to laboratory screening.	While the first key message seemed to have looked over-reassuring as the future of the virus was impossible to predict, the second one, however, was consistent with the official message indicating that the involved authorities were in agreement.
